# Automatic and adaptive heterogeneous refractive index compensation for light-sheet microscopy

**DOI:** 10.1038/s41467-017-00514-7

**Published:** 2017-09-20

**Authors:** Duncan P. Ryan, Elizabeth A. Gould, Gregory J. Seedorf, Omid Masihzadeh, Steven H. Abman, Sukumar Vijayaraghavan, Wendy B. Macklin, Diego Restrepo, Douglas P. Shepherd

**Affiliations:** 1 Department of Physics, Colorado State University, Fort Collins, CO 80523 USA; 2 Department of Cell and Developmental Biology, University of Colorado Anschutz Medical Campus, Aurora, CO, 80045 USA; 3 Department of Pediatrics, University of Colorado School of Medicine, Aurora, CO, 80045 USA; 4 Pediatric Heart Lung Center, University of Colorado Anschutz Medical Campus, Aurora, CO, 80045 USA; 5 Department of Ophthalmology, University of Colorado Anschutz Medical Campus, Aurora, CO, 80045 USA; 6 Department of Physiology and Biophysics, University of Colorado Anschutz Medical Campus, Aurora, CO 80045 USA; 7 Department of Physics, University of Colorado Denver, Denver, CO 80217 USA

## Abstract

Optical tissue clearing has revolutionized researchers’ ability to perform fluorescent measurements of molecules, cells, and structures within intact tissue. One common complication to all optically cleared tissue is a spatially heterogeneous refractive index, leading to light scattering and first-order defocus. We designed C-DSLM (cleared tissue digital scanned light-sheet microscopy) as a low-cost method intended to automatically generate in-focus images of cleared tissue. We demonstrate the flexibility and power of C-DSLM by quantifying fluorescent features in tissue from multiple animal models using refractive index matched and mismatched microscope objectives. This includes a unique measurement of myelin tracks within intact tissue using an endogenous fluorescent reporter where typical clearing approaches render such structures difficult to image. For all measurements, we provide independent verification using standard serial tissue sectioning and quantification methods. Paired with advancements in volumetric image processing, C-DSLM provides a robust methodology to quantify sub-micron features within large tissue sections.

## Abstract (web summary)

Optical clearing of tissue has enabled optical imaging deeper into tissue due to significantly reduced light scattering. Here, Ryan *et al*. tackle first-order defocus, an artefact of a non-uniform refractive index, extending light-sheet microscopy to partially cleared samples.

## Introduction

A rapid expansion in the number of combinatorial techniques for quantifying the spatiotemporal distribution of biological signaling and connectivity in intact tissues has occurred in recent years. The parallel development of novel fluorescence labeling, preparation methods that render tissues optically clear, and imaging instrumentation has enabled the detailed study of molecules, cells, and structures within large biological samples. Many techniques have emerged to render intact tissue optically transparent, each with different final tissue properties such as the refractive index (RI), de-colorization, RNA accessibility, protein accessibility, retention of native morphology,and endogenous fluorophore compatibility^1–3^.

A natural technique for quantitiative flourescent measurements of large volumes of intact tissue and model animals is light-sheet fluorescence microscopy (LSFM). LSFM is capable of selective excitation of fluorophores within a thin area of a specimen, limiting signal degrading out-of-focus light and undesired photo bleaching^4, 5^. In contrast, conventional wide-field microscopy or laser-scanning confocal microscopy excite across the entire volume of a sample during the imaging of any single plane, generating large background signals a﻿n﻿d photoblea﻿chi﻿ng. Laser-scanning two-photon microscopy reduces background and photobleaching due to out-of-focus excitation, but is limited in throughput and available fluorophores^6^. For optically transparent samples, LSFM has significant advantages over point-scanning techniques because it combines optical sectioni﻿ng with minimal photo bleaching and superior acquisition speed^7^. Despite these advantages, current LSFM implementations do have limitations when applied to optically cleared tissue. Because LSFM must maintain co-planar excitation and detection planes for sharp imaging, the majority of LSFM designs suffer image degradation due to first-order defocus. Royer et al.^8^ recently highlighted this problem in high-resolution measurements of developing embryos using LSFM. First-order defocus is even more problematic when imaging large optically cleared tissue because the inherent RI variability and large optical path lengths result in gross misalignment of the excitation and detection planes. This Places restrictions on usable optics, resolution, imaging depth, and sample preparations^8, 9^.

Motivated by existing work on remote focusing and self-adapting LSFM designs^8, 10–13^, we developed C-DSLM (cleared tissue digital scanned light-sheet microscopy). This design is optimized to automatically account for spatially heterogeneous RI in thick optically cleared samples without the need for user intervention or a heavy investment in specialized optical/optomechanical components such as piezo stages, adaptive optics, or microscope objectives. In existing LSFM configurations, RI correction is typically achieved by changing the position of the detection objective using a piezo stage or changing the axial position of the exciting light-sheet using a galvanometer mirror. Here, we demonstrate that without dynamic calibration this approach leads to first-order defocus and loss of image quality as the axial distance increases from the initial calibration point. Using C-DSLM and recent advancements in volumetric image processing, we quantify individual oligodendrocytes cells, myelin tracks, and distal lung complexity. We show that C-DSLM enables volumetric quantification in tissue that is intentionally under-cleared to retain a lipid-bound endogenous reporter of myelin. We demonstrate that C-DSLM provides first-order defocus correction for a range of microscope objectives. Finally, we independently verify each measurement using uncleared tissue, serial sectioning, and standard microscopy techniques that in contrast to C-DSLM, require a significant time investment to obtain a single measurement.

## Results

### Microscope development

**Fig. 1:**
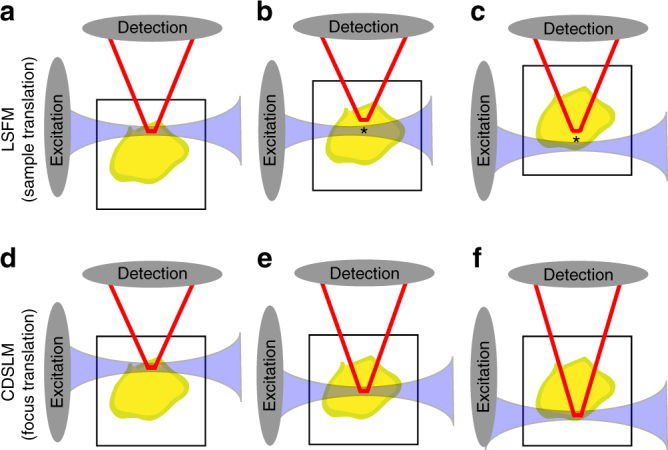
First-order defocus in light-sheet fluorescence microscopy (LSFM). Standard LSFM without compensation begins with co-planar light-sheet and detection planes. At the surface, there is minimal first-order defocus (**a**). The sample is then physically translated to generate volumetric images (**b** and **c**). As the path length increases, first-order defocus causes the excitation or detection focal planes to move closer to the optical element by approximately *n × *Δ*z* (* in ***﻿﻿b﻿﻿*** and ***c***), where *n* is the local refractive index (RI) and Δ*z* ﻿is the ﻿excitation or detection pathway optical path length. This displacement will vary based on the spatial location within optically cleared tissue and the RI mismatch. While newly available RI matched objectives minimize this RI mismatch, these objectives have narrow depth-of-fields due to high-numerical apertures. Therefore, these objectives are more susceptible to first-order defocus. Previous compensation methods for stage translation LSFM with or without RI corrected optics have included large depth-of-field detection^14^, physical translation of the excitation and detection objective^15, 16^, or extended depth-of-field imaging ^16, 17^. Cleared tissue digital scanned light microscopy (C-DSLM) compensates for first-order defocus created by such RI heterogeneity through independent positioning of the excitation focus, detection focus, and lateral position of the light-sheet using computational algorithms and electro-tunable lenses. C-DSLM holds the sample stationary while determining the optimal combination of these three degrees of freedom to optimize over an entire image stack (**d**-**f**). The axial position of the light-sheet, determined by the galvanometer mirror position, provides a constant reference across different imaging conditions, as only those fluorophores within the light-sheet are excited

To create an automated and autofocusing light-sheet microscope, we utilized electro-tunable lenses (ETL) inserted in both the excitation and detection arms of a digital scanned light-sheet microscope (Fig. 1, Supplementary Fig. 1, and Supplementary Notes 1–3). In this configuration, the position of the exciting light-sheet was decoupled from the imaging plane and can be positioned independently to correct for heterogenous RIs. We paired these ETLs with microcontroller-based control of galvanometer mirrors, laser modulation, and an automated stage to create a microscope that provided semi-automated imaging of cleared thick tissue.

To autofocus in heterogenous RI samples, we generated a sinusoidal reference pattern on the excitation light-sheet to establish a known feature for determining the location of the light-sheet. We identified the focal plane in which the emitted fluorescence best matched the given sinusoidal modulation frequency by sweeping the detection ETL (ETL-2) around a user-specified estimate of the focal plane. At set imaging focal planes around the current light-sheet position the pattern was imaged. The pattern frequency was ﻿then estimated using a Fast Fourier Transform (FFT). The mean squared error of the calculated frequency with respect to the known frequency of the pattern was calculated for each image plane. The imaging plane with minimum ﻿mean squared error (MSE) provide﻿d an initial rough estimate of the correct focal plane. To refine our estimate of the imaging plane, we then calculate﻿d the Shannon Entropy of the Discrete Cosine Transform (SE-DCT) for a number of planes around the FFT selected plane. T﻿he SE-DCT ﻿enable﻿d the determination of the optimal focal plane given the aberrations of the current optical configuration (see Methods section)^8^. Even in highly scattering thick samples, this approach was robust as the frequency of the patterned light-sheet could be lowered until a reasonable confidence interval was found to determine an initial starting point. This approach lowered computation time and the number of full exposure images required, as the initial focal plane search utilized FFTs and low laser power then switched to full plane imaging and the more costly SE-DCT.

An additional benefit of patterning the exciting light-sheet was the ability to implement HiLo reconstruction, a structured illumination technique that utilizes one patterned image and one uniform image to reconstruct the in-focus fluorescence in Fourier space^18^. This approach was more resistant to scattering than other structured light-sheet techniques because it does not rely on precise and uniform relative shifts in the excitation pattern across the entire imaging area^15, 18^.

This approach does not translate the sample or objectives during an axial imaging scan, requiring minimal physical restraint as compared to other cleared tissue LSFM approaches, which involve rigid physical immobilization of the sample due to mechanical translation of the sample or objectives^1, 8, 16^.

### Imaging at depth within heterogeneously cleared samples

Myelin is essential for proper neural function and demyelination or dysmyelination underlie a variety of brain disorders^19, 20^ Because of the lipid-rich composition, it is a poor candidate for use with tissue clearing techniques that work through lipid removal. We hypothesized that by intentionally under-clearing mouse brains, we would retain a lipid-bound protein coupled to an endogenous fluorescence reporter of myelinated structures. To visualize oligodendrocytes, we chose to use transgenic mice expressing eGFP under the control of the myelin proteolipid-promoter (PLP-eGFP^21, 22^). PLP-eGFP mice have been used previously to characterize oligodendrocyte morphology, due to the presence of high eGFP expression in the cytoplasm of mature oligodendrocytes rendering cell bodies and cytoplasmic domains within myelin sheaths visible^22^. We prepared samples using both advanced CLARITY and PACT, reducing the exposure to the SDS solution in each case^1, 16^. Using advanced CLARITY, we repeatedly found that the endogenous PLP-eGFP was removed, while under-cleared PACT retained PLP-eGFP fluorescence. We verified this is due to PLP-eGFP being washed out during the clearing process, not due to quenching of the fluorescence by labeling with anti-GFP antibodies. While it was possible to under-clear using advanced CLARITY or utilize other clearing techniques, we have found in practice that PACT provides a much greater degree of control of the clearing parameters and tissue stability^1, 2, 3, 16, 23^.

**Fig. 2:**
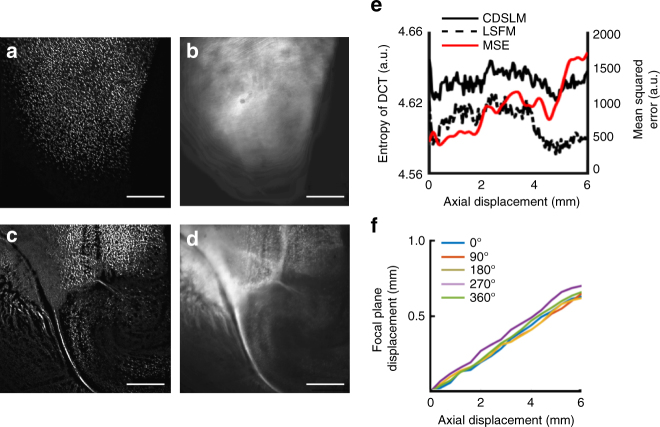
Quantification of signal-to-noise enhancement from cleared tissue. Cleared tissue digital scanned light microscopy (C-DSLM) was implemented with a ×4, NA 0.2 detection objective. The light-sheet full-width at half-maximum (FWHM) was adjusted to ∼16 μm to yield an approximate Rayleigh length of 1600 μm before Gaussian﻿ beam scanning. Each axial step was ∼3 μm. **a**−**d** C-DSLM **a**, **c** and standard light-sheet fluorescence microscopy (LSFM) **b**, **d** imaging of individual focal planes 1 mm (top-**a**, **b**) and 6 mm **c**, **d** into the stack. C-DSLM clearly delineated individual oligodendrocyte cells and myelin tracks that were either out-of-focus or blurred due to first-order defocus in standard LSFM (*scale bars*—500 μm). **e** Quantification of Shannon Entropy of the Discrete Cosine Transform (SE-DCT) for C-DSLM and standard LSFM as well as mean squared error (MSE) comparison between C-DSLM and standard LSFM throughout the entire image stack. **f** Quantification of first-order defocus in standard LSFM as a function of axial imaging depth. The sample was imaged at 0°, 90°, 180°, 270°, and 360° fixed rotations. Representative of *n* = 10 experiments. All imaging was done within the cortex or olfactory bulb of﻿ ﻿pa﻿ssive CLARITY (PACT) cleared proteolipid-promoter eGFP (PLP-eGFP) mouse ﻿tissue﻿

To demonstrate the advantages of the first-order defocus correction we implemented in C-DSLM, we imaged PLP-eGFP mouse brain tissue using C-DSLM and standard LSFM methods. C-DSLM images were acquired using our semi-automated imaging method (see Methods section). Standard LSFM images were acquired by aligning the imaging plane of the objective with zero applied current to ETL-2 with the neutral position of the exciting light-sheet. The sample positioning stage was used to position the edge of the tissue into the light-sheet. Images were acquired by translating the sample through the stationary exciting light-sheet using a piezo stage. After acquiring the standard LSFM image, we then calculated the required first-order defocus correction by positioning the sample using the piezo stage and recording the required focal plane displacement to bring the image into focus. (Fig. 2a–d). Additionally, we calculated the SE-DCT for both C-DSLM and standard LSFM (Fig. 2e). SE-DCT was one potential metric of image quality that we and others have utilized (see Methods section). Because the SE-DCT is a frequency-based technique, it is particularly suited to determining image quality when low frequency components dominate the image^8^. C-DSLM consistently displayed a higher SE-DCT throughout the focal stack. Additionally, at each image plane we calculated the MSE between C-DSLM and standard LSFM. We found that it steadily increases as imaging depth, and therefore first-order defocus, increases.

We repeated this measurement for 0, 90, 180, 270, and 360 degree sample rotation, verifying that accounting for the axial distance of RI matching media and sample was critical for determining the correct relative positioning of the light-sheet and detection focal plane (Fig. 2f).

**Fig. 3:**
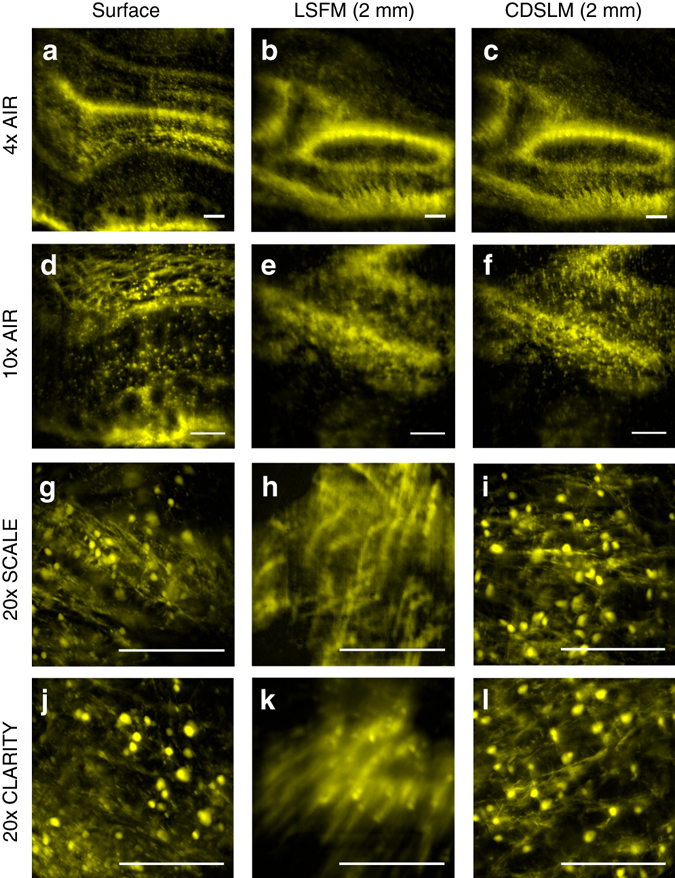
First-order defocus correction. Images were acquired using **a**−**c** ×4, NA 0.2, refractive index (RI) = 1.0; **d**−**f** ×10, NA 0.28, RI = 1.0; **g**−**i** ×20, NA 1.0, RI = 1.38; and **j**−**l** ×20, NA 1.0, RI = 1.45 detection objectives. All images were obtained from the same passive CLARITY (PACT) cleared proteolipid-promoter eGFP (PLP-eGFP) olfactory bulb region. The light-sheet full-width at half-maximum (FWHM) and excitation electro-tunable lens (ETL-1) limits for Gaussian beam scanning were adjusted to yield the best signal-to-noise for individual objectives. Cleared tissue digital scanned light-sheet microscopy ﻿(C-DSLM) clearly delineated individual oligodendrocyte cells and myelin tracks that were either out-of-focus or blurred due to first-order defocus in standard light-sheet fluorescence microscopy (LSFM) for all objectives. Because of differences in RI environment and depth-of-field for individual objectives, the magnitude of the first-order defocus differs in each case. (all *scale bars*—250 μm)

We then repeated this measurement using a variety of RI-matched and RI-mismatched objectives (Fig. 3). The need for first-order defocus correction becomes more critical as the depth-of-field of the detection objective decreases. Additionally, we found that the small mismatch in RI for the SCALE (RI = 1.38) objective as compared to the CLARITY (RI = 1.45) objective leads to a larger first-order defocus and limits the effective scan range of C-DSLM.

**Fig. 4:**
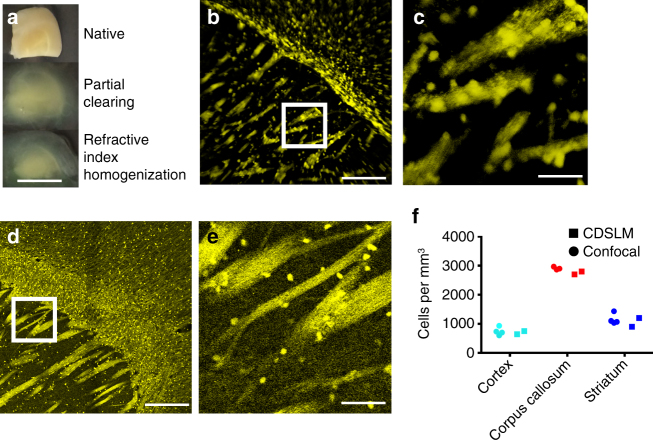
Cleared tissue digital scanned light-sheet microscopy (C-DSLM) imaging of proteolipid-promoter eGFP (PLP-eGFP) mouse brain. **a** PLP-eGFP mouse brain sections were passive CLARITY (PACT) cleared and refractive index (RI) homogenized with intentional under-clearing to retain myelinated structures (*scale bar*—1 mm). **b** Individual image plane within PLP-eGFP mouse cortex cord from a 1.3 mm × 1.3 mm × 5 mm axial image (*scale bar*—250 μm) acquired using the ×10,NA 0.28 detection objective. The light-sheet full-width at half-maximum (FWHM) was adjusted to ∼10 μm to yield an approximate Rayleigh length of 700 μm before Gaussian beam scanning. Each axial step was ∼1 μm. Supplementary Movie 1 shows a volumetric rendering of this region. **c** Expanded image of highlighted area (*white box*) in **b**. Individual cells, dendrites, and pencil fibers are clearly visible (*scale bar*—50 μm) **d** Tiled confocal image of individual 30 μm serial section of PLP-eGFP mouse cortex imaged using confocal microscopy (×20, NA 0.95 water-immersion objective) (*scale bar*—250 μm). **e** Expanded image of highlighted area (*white box*) in **d**. Individual cell bodies, dendrites, and pencil fibers are clearly visible (*scale bar*—50 μm). **f** Verification of C-DSLM PLP-eGFP+ cell counting. Serial sectioning of uncleared PLP-eGFP cortex tissue measured by confocal microscopy (*n* = 4 animals, 100 individual image planes per animal) versus cleared and intact PLP-eGFP cortex tissue measured by C-DSLM (*n* = 2, 6000 individual images planes per animal)

**Fig. 5:**
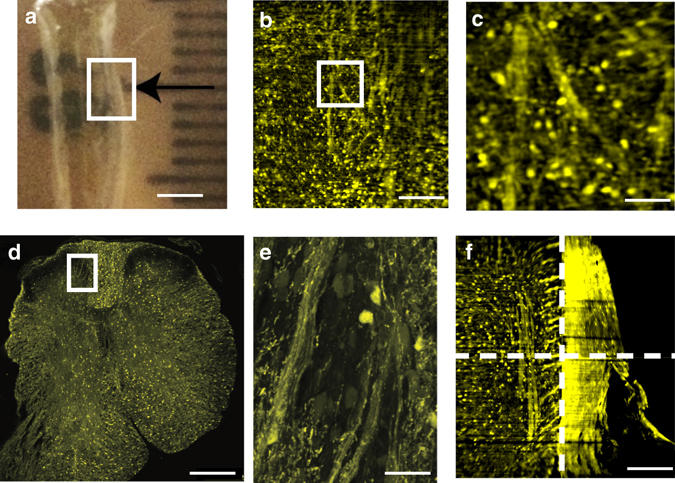
Cleared tissue digital scanned light-sheet microscopy (C-DSLM) imaging of proteolipid-promoter eGFP (PLP-eGFP) mouse spinal cord. **a** PLP-eGFP mouse spinal cords were passive CLARIT﻿Y﻿ (PACT) cleared in the same manner as PLP-eGFP brain sections. Each *black tick* of the ruler is 1 mm. Area tiled using C-DSLM is shown by the *white box*. (*scale bar*—2.5 mm) **b** Individual image plane within PLP-eGFP mouse spinal cord from a 1.3 mm × 1.3 mm × 6 mm axial image (*scale bar*—250 μm) acquired using ×10, NA 0.28 detection objective. The light-sheet full-width at half-maximum (FWHM) was adjusted to ∼10 μm to yield an approximate Rayleigh length of 700 μm before Gaussian beam scanning. Each axial step was ∼1 μm. **c** Expanded image of highlighted area (*white box*) in **b** showing individual cells and myelin fibers (*scale bar*—50 μm). Supplementary Movie 2 shows a volumetric rendering of this region. **d** Tiled confocal image of 30 μm axial serial section of uncleared PLP-eGFP spinal cord (×20, NA 0.95 water-immersion objective) (*scale bar*—250 μm). **e** Expanded view of highlighted area (*white box*) in **d** showing individual cells and myelin fibers (*scale bar*—50 μm). **f** To investigate large-scale network connectedness we tiled images over a 4 mm × 3 mm in-plane region, 5 mm out-out-plane region, with individual images (1.3 mm × 1.3 mm in-plane, 5 mm out-of-plane) overlapping images by 25% in each area. *Dashed lines* delineate independent volumes used for final tiled image (*scale bar*—100 μm). Supplementary Fig. 2 shows network identification in this tiled volume. Supplementary Movie 3 shows volumetric rendering o﻿f﻿ the tiled image in **f**, well matching the confocal image presented in **d**. Supplementary Movie 4 shows a plane-by-plane rendering of the image in **c**. These images and analyses are representative of five experiments for spinal cords isolated from different animals

After verifying that C-DSLM automatically corrected for first-order defocus due to heterogeneous RI in under-cleared PLP-eGFP tissue, we turned to quantifying individual cells and myelin tracts in PACT-cleared PLP-eGFP mouse brains (Fig. 4) and PACT-cleared PLP-eGFP mouse spinal cord (Fig. 5). After partially clearing tissue (Figs. 4a and 5a), we utilized C-DSLM to image individual PLP-eGFP cells and myelin tracks (Figs. 4b, c and 5b, c, Supplementary Movies 1–2). We independently verified the accuracy of C-DSLM PLP-eGFP+ cell counts in the cortex using 30 μm serial sections and confocal microscopy (Figs. 4d–f and 5d, e). We additionally imaged multiple adjacent areas with 20% overlap and re-assembled a large-scale tiled image of the PLP-eGFP spinal cord to ensure that we could track long distance myelin fibers (Fig. 5f, Supplementary Fig. 2, Supplementary Movies 3–4).

### Multi-color histology of intact samples

To demonstrate the ability of C-DSLM to maintain multiple colors co-planar during volumetric imaging, we utilized adult rat lungs. Distal lung development occurs at late gestational age in human fetuses and is sensitive to antenatal and postnatal stressors^24^. For example, many premature birth infants develop bronchopulmonary dysplasia (BPD), the chronic lung disease of prematurity due to many factors such as ventilation, infection and a change in oxygen environment^25, 26^. Newborn rats are commonly used to study human infant distal lung development because the rat distal lung continues to develop after birth, providing a robust model to study the effects of these antenatal and postnatal stressors^27^. In contrast to the adoption of advanced fluorescence imaging methods to quantify molecules, cells, and structures within the neuroscience community, distal lung structure quantification still heavily relies on traditional two-dimensional histology measurements to quantify airway and vascular networks^28, 29^. This methodology samples only a small portion of the distal lung, lacks information on spatial distribution of key signaling proteins, and is potentially error-prone due to time consuming physical serial sectioning of thin tissue sections to determine lung complexity.

**Fig. 6:**
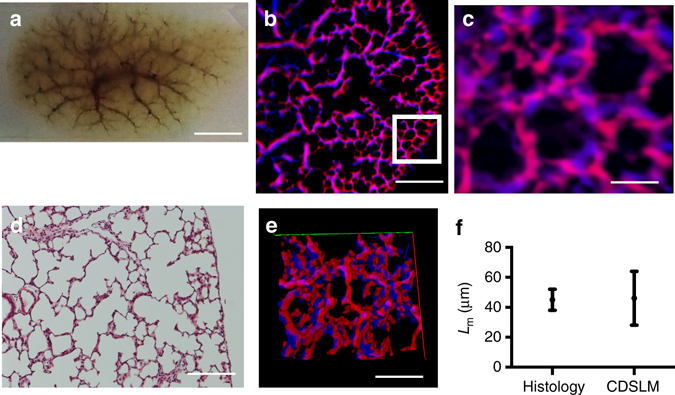
Cleared tissue digital scanned light-sheet microscopy (C-DSLM) imaging of rat lungs. **a** Rat lungs (day 14) were collected, inflated, fixed, cleared using passive CLARITY (PACT) (*scale bar*—5 mm). **b** Individual image plane of epithelium (*blue*) and endothelium (*red*) within rat lung from a 1.3 mm × 1.3 mm × 6 mm axial image (*scale bar*—250 μm) acquired using the ×10, NA 0.28 detection objective. (*scale bar*—250 μm) The light-sheet full-width at half-maximum (FWHM) was adjusted to ∼10 μm to yield an approximate Rayleigh length of 700 μm before Gaussian beam scanning. Each axial step was ∼1 μm. **c** Expanded image of highlighted area (*white box*) in **b** showing distal lung structure. Alveolar structures were clearly marked by both airway (*blue*), blood vessels (*red*), and overlap between the two networks (*magenta*) (*scale bar*—50 μm). **d** 2D histology of distal lung imaged using white light and a ×20 air immersion objective (*scale bar*—200 μm). **e** 3D reconstruction of c. The true complexity of the distal lung was lost in traditional histology but can be clearly seen using tissue clearing and light-sheet imaging. Supplementary Movie 5 shows a volumetric rendering of this region. **f** Verification of C-DSLM distal lung results using Mean Linear Intercept (MLI) to determine the chord length (*L*_*m*_). We created a new 3D MLI algorithm that imposes a three-dimensional grid (vs the traditional lines for planar MLI) and calculates nearest-neighbor intersections across the volume measurement. Serial sectioning and histological staining measured by wide-field microscopy (*n* = 23 individual histology slices) versus cleared and intact lung lobe measured by C-DSLM (*n* = 5 entire lungs, 5000 individual image planes). *Error bars* are standard deviation (SD). Mean *L*_m_ values for histology and C-DSLM *L*_m_ did not significantly differ. The larger C-DSLM SD was due to both our inclusion of 3D information and proximal lung blood vessels, leading to a larger spread in calculated *L*_m_

We utilized C-DSLM to image individual PACT-cleared newborn rat lung lobes (day 14) labeled using an endothelial cell marker (Isolectin B4-Alexa647) and an epithelium marker (Cytokeratin-Alex488). Our standard protocol was to remove the blood via heparin and perfusion, however we retained the blood for Fig. 6a to illustrate the integrity of the entire vascular tree after PACT-clearing. Using C-DSLM we imaged the vascular and airway networks within an entire PACT-cleared lung lobe at a voxel size of 0.65 × 0.65 × 1.5 μm (Fig. 6b, c). We verified that C-DSLM produces the same distal lung structure as histological sectioning by creating a three-dimensional (3D) variant of mean linear intercept (MLI) analysis^30^. MLI is one of the standard metrics utilized to characterize lung complexity. We verified that our 3D MLI metric matched standard two-dimensional MLI using histologic sections from non-cleared day 14 rat lungs (Fig. 6d, f). Critically, C-DSLM captured 3D morphology lost in serial sectioning (Fig. 6e, Supplementary Movie 5).

## Discussion

LSFM is a promising technology to pair with optically cleared tissue, but first-order defocus and other optical aberration have limited its use to specialized microscope designs, limited axial depth, or samples rendered completely clear using techniques that alter the tissue morphology or fluorophore compatibility. Here, we demonstrated C-DSLM, a LSFM design that accounts for first-order defocus and background fluorescence due to spatial heterogeneous RI in thick tissue samples that have been optically cleared. The flexible design, based on computational autofocusing with the addition of two electro-tunable lenses and two relay lenses to a standard DSLM, enabled imaging of a variety of biochemical clearing and labeling techniques.

ETLs have previously been utilized in either the excitation and detection arm of LSFM designs. In one of the first demonstrations, Fahrbach et al.^10^ achieved > 700 fps imaging of a beating zebrafish heart by driving a detection arm ETL at high frequencies. Their work utilized a excitation mirror and ETL pre-synchronization scheme that relied on a nearly uniform RI and a physically restrained zebrafish^10, 11^. C-DSLM similarly utilizes an ETL in the detection pathway, but was designed for dynamic and automated alignment of the exciting light-sheet and detection focal plane using a FFT and SE-DCT based algorithm.

Chmielewski et al.^12^ addressed the distortion of the exciting light-sheet in weakly scattering samples by utilizing two ETL lenses in the excitation arm to optimize the thickness of the sheet for both *Drosophila* and zebrafish embryos. However, their work still relied on a piezo to translate the excitation objective and was focused on reduction of scattering in relatively thin samples. Hedde and Gratton^31^ demonstrated that an ETL can replace the fast-axis galvanometer mirror in a DSLM, but still relied on physical translation of the sample for axial scanning. C-DSLM similarly utilizes a ETL in the excitation pathway, but rapidly oscillates the ETL through automatically determined limits. This eliminates shadowing via altering the entrance angle of the light-sheet into the sample and provides higher contrast through Gaussian beam scanning^32–34^. The adoption of a similar multi-ETL excitation system as Chmielewski et al.^12^ may further benefit C-DSLM for high-NA objectives. Because C-DSLM does not utilize objective or sample piezo stages, it is currently the only light-sheet imaging technique to utilize tunable optical elements within both the detection and excitation arm to automatically account for optical aberrations.

It is possible that the RI will vary within a single imaging plane such that a uniform offset across the image plane is not sufficient. For low- to moderate-NA objectives this variation is likely smaller than the depth-of-field. For high-NA objectives this variation may exceed the depth-of-field. In this scenario, it would be possible to split the image plane into multiple sections and use C-DSLM to generate multiple images that span the focus across the image area. Another option would be to implement extended depth-of-field imaging. One previous approach was﻿ to rapidly oscillate the detection lens to capture multiple focal planes at once^17^ and another was the computationally expensive scheme utilized in SPED^35^. While SPED exploited the spherical aberrations found in many LSFM designs, other groups have sought to minimize or computationally account for optical aberrations. For example, Masson et al.^36^ addressed the scattering of emitted fluorescence using a computational model of the pupil function, under the unrealistic assumption of homogeneous RI in a CUBIC cleared sample. Paired with their computational scheme, Masson et al.^36^ introduced an alternate clearing procedure that more closely matches the RI of water-immersion objectives to ensure light-sheet compatability.

CLARITY optimized light-sheet microscopy (COLM)^16^ and other cleared tissue LSFM designs have addressed, but not explicitly quantified, the focal drift due to this spatial varying RI. The authors of COLM note that both focal plane and light-sheet centering offsets must be determined before imaging throughout the entirety of a Thy1-YFP brain, even with a specialized RI matching objective, but did not provide quantitative evaluation of these offsets or a dynamic, automated calibration process^16^. C-DSLM offers a rapid, flexible, and adaptable methodology to automatically maintain co-planarity of the detection and excitation planes using fast ETLs and image based autofocusing. C-DSLM works across a wide variety of optically cleared tissue sample preparations. This was demonstrated by our unique quantification of individual oligodendrocytes and myelin tracks using under-cleared PLP-eGFP tissue. C-DSLM has the additional benefit of holding samples stationary, enabling imaging of fragile structures, such as intact retinas^37^.

Autopilot addressed the issues of heterogeneous RI, scattering, and light-sheet shaping for high-resolution time-lapse measurements of developing embryos^8^. This work clearly demonstrated the need for first-order defocus compensation, finding that even the small differences in RI between organelles within a developing embryo will lead to image blurring. There were many advancements within the Autopilot framework beyond accounting for first-order defocus, such as characterizing and adapting the exciting light-sheet to best match the sample properties to maximize image quality. Adapting these ideas to thick tissue samples using adaptive optics to control the shape of the exciting light-sheet is a clear next step in this area.

As tissue clearing techniques continue to improve and enable robust spatial quantification of RNA, protein, cells, and structures, a broadly compatible LSFM platform will be necessary. Affordable and simple to implement, C-DSLM can handle multiple clearing methods and is easily combined with other LSFM advancements to provide dynamic autofocusing in samples with a heterogeneous RI.

## Methods

### C-DSLM optical layout

The excitation arm consisted of a multi-color laser source (Coherent OBIS 488 nm, 532 nm diode laser, and Coherent OBIS 640 nm; Thorlabs RGB46HA), fiber collimator (FC–Thorlabs TC06FC-633), ETL (ETL-1—Optotune EL-10-30), pair of galvanometer mirrors (2D-galvo—Thorlabs GVSM002), scan lens (SL—Thorlabs CLS-SL), tube lens (TL1—Nikon MXA22018), and objective (EO)—a ×5, 0.14 NA, 33.5 mm WD (Edmund Optics 59-876) and a ×10, 0.28 NA, 33.5 mm WD (Edmund Optics 59-877) have been demonstrated in this work—to create the sheet and displace the beam along the optical axis of the detection arm. The galvanometer mirrors and camera were controlled by an Arduino Uno unit (Sparkfun DEV-11021) with three PowerShield DAC boards (Visgence, Inc.) while laser intensity was controlled by an Arduino Uno unit (Sparkfun DEV-11021) with three PowerShield DAC boards (Visgence, Inc.).

To generate the light-sheet, the scanning mirror corresponding to motion perpendicular to the imaging plane (fast-axis) was swept once per image plane. The second scanning mirror positioned the light-sheet along the detection axis. ETL-1 optimized alignment of each laser source, enabled fine-tuning of the excitation arm focus within the sample, Gaussian beam scanning, and allowed greater flexibility for the sample position within the imaging chamber.

A 3D-printed sample chamber, with glued #1 quartz coverslips (SPI Supplies 01019T-AB), was mounted to a manual rotation stage (Thorlabs PR01) and centered at the focal length of both the excitation and detection objectives such that the full range of excitation beam scanning was available (∼6 mm with the 5x EO). Two stage configurations were utilized: an automated micrometer-driven *xyz* stage controlled using OpenStage hardware and software was utilized for C-DSLM imaging and tiling^38^ or an automated piezo-driven stage (Newport M-423, Newport 8303, Newport 8742) and an *xyz* manual stage (TS—Thorlabs PT3) was utilized for comparison of C-DSLM to stage-scanning LSFM. Both configurations position a sample rotation stage/sample mount (RSSM) consisting of an Arduino Uno controlled stepper motor (Sparkfun DEV-11021, Sparkfun ROB-09238) and rigid-mounted 200 μl pipette tip, within the imaging chamber.

The detection arm accommodated a ×4, 0.2 NA, WD–17.2 mm (Nikon MRH00041), ×10, 0.28 NA, 33.5 mm WD (Edmund Optics 59-877), ×50, 0.55 NA, 18.1 mm WD (Edmund Optics 59-881), ×20, 1.0 NA, 5.6 mm WD, RI = 1.38 (Zeiss 421459-9770), or ×20, 1.0 NA, 5.6 mm WD, RI = 1.45 (Zeiss 421459-9970) objective (DO) using a non-rotating translatable objective mount (OM—Thorlabs SM1NR1). A tube lens (TL2—Nikon MXA22018 or Thorlabs TTL165-A), adjustable periscope mirror (M1—Thorlabs BB1-E02), ETL (ETL-2—Optotune EL-16-40-TC) placed within a *4f* system (×2 L1—Thorlabs AC254-300-A), adjustable periscope mirror (M2—Thorlabs BB1-E02), automated filter wheel (FW—Thorlabs FW102C), and sCMOS (Hamamatsu C11440-22CU) camera completed the detection optics. In a *4 f* configuration the focus of the detection arm can be swept while maintaining telecentricity.

### System characterization

The initial alignment of the system is described in Supplementary Note 3. After alignment, we utilized solutions of fluorescent dye (Alexa Fluor 488, ThermoFisher A20000, Alexa Fluor 532, ThermoFisher A20001, and Alexa Fluor 647, ThermoFisher A20006) to align and then characterize the width of the light-sheet for the 488 nm, 532 nm, and 640 nm laser sources. Following this step, we characterized the point-spread function (PSF) of the system and chromatic aberration utilizing fluorescent microspheres (TetraSpeck, ThermoFisher T7280, for PSF quantification and FocalCheck, ThermoFisher F7237, for chromatic aberration) dispersed in a mix of RI matching solution (RIMS) and 1% agar (Supplementary Fig. 3).

We additionally verified that ETL-2 did not affect the image properties of the system by removing ETL-2 and re-imaging the bead sample using the automated piezo-driven stage. We found that if ETL-2 was not perfectly flat and at the correct *4f* position, it may introduce a change in focal length across the field-of-view (Supplementary Fig. 4), as previously observed by Fahrbach et al.^10^. Careful iteration of the *4f* alignment procedure (Supplementary Note 3), leveling of ETL-2, and ensuring excess pressure was not placed on ETL-2 by retention rings were all necessary to alleviate this issue.

### Independent axial positioning

To compensate for first-order defocus, the light-sheet and focus must move independently. The axial placement of the light-sheet was set by one axis of the 2D galvanometer mirror. The detection focus was controlled by ETL-2 which shifted the focus by Δ*z*_ETL-2_ (Supplementary Note 2). This displacement was determined by FFT and SE-DCT image based autofocusing routine or manual calibration.

### Volumetric imaging using C-DSLM method

A 3D scan with the C-DSLM method involved stepping the light-sheet and imaging plane though axial slices of the sample volume and acquiring an image at each slice while rapidly oscillating ETL-1 for Gaussian beam scanning^31, 33, 34^ and to introduce a slight tilt to the light-sheet^32^. This tilt mitigates the effect of shadowing by altering the entrance angle on a given excitation line. An aperture at the back of the excitation objective may be manually adjusted to match the Rayleigh length of the exciting light-sheet to the width of imaging area. With a simple calibration process that maintains the co-alignment of the detection and excitation planes, a wide variety of sample preparations are available to the technique. While a general sample preparation calibration can be done prior to imaging a sample of interest, the power of C-DSLM lies in sample- and sub region specific corrections to the calibration, as this produced the clearest images. Calibration was performed by determining the best focus (using automated or manual methods) for both the excitation and imaging pathways at various light-sheet positions (depending on the complexity and degree of heterogeneity of the sample, 4–10 positions may be required) and creating a reference curve of the co-planar positions that was later used during a full imaging scan.

### Automatic first-order defocus correction

Several images at nearby focal offsets were acquired for each light-sheet position. The focus was found by first determining the focal plane that best matched the known HiLo pattern by calculating the mean squared error of the obtained frequency with respect t﻿o the known frequency. We then maximized the SE-DCT within a local region surrounding this initial guess to determine the best focal plane. After the best focal plane was selected, three pixel rows (far left, center, and far right of image) that were orthogonal to the excitation propagation direction were summed during a sweep of ETL-1 around the initially user set value to determine the limits of ETL-1 oscillation. A robust discussion supporting the use of the SE-DCT for autofocusing can be found in Royer et al.^8^. In addition to this detailed comparison of many possible image quality metrics, Royer et al.^8^ determined that normalizing the SE-DCT and applying appropriate low-pass filtering optimized the use of the SE-DCT as an image quality metric for light-sheet microscopy. The typical image depth in the Autopilot framework was 100 μm. In contrast, the typical image depth in C-DSLM was greater than 3 mm and first-order defocus was the main contributor to loss of image quality. We found in the C-DSLM context that normalization was not a critical as minimal median filtering (3 × 3 pixels for the ×10 NA 0.28 DO). It was also possible to utilize the SE-DCT to conduct the initial search using the patterned beam. In practice, we found the additional computational cost as compared to identifying the principle frequency using FFT did not provide a reasonable increase in rough alignment of the light-sheet and imaging plane.

### Manual first-order defocus correction

The user selected a light-sheet plane and adjusts the focus of both ETLs to obtain the sharpest image as judged by the user. This process ﻿was repeated for multiple image planes throughout the desired axial imaging depth. The calibration curve was then interpolated during a full scan to obtain the sample-specific corrections. This process is subjective, but necessary to determine the ground truth focal plane and verify the autofocusing routine.

A complete and unique calibration was obtained by performing either focusing method. For automatic focusing, the autofocus routine was run at user-specified intervals throughout the axial stack to create a limited number of calibrated focal planes before imaging proceeded. For both automatic focusing and manual calibration, a linear interpolation at the Nyquist limit was created between each calibration point for a unique map of ETL-1, ETL-2, and the light-sheet position. Imaging then proceeded at the appropriate axial step size for the given optical configuration. At each axial position, the Hamamatsu C11440-22CU was run in light-sheet mode, with the digital slit position corresponding to the in-plane location of the exciting Gaussian beam. The beam was swept once for uniform and again for HiLo. The mirror control Arduino Uno controlled the speed of the galvanometer mirror sweep, start trigger for the Hamamatsu C11440-22CU light-sheet mode, and activation of the laser. During the HiLo sweep, the laser was modulated by the mirror Arduino Uno through hardware interrupt of the laser Arduino Uno. The digital slit width was twice the spatial spacing of the HiLo pattern, ensuring that the necessary spatial and frequency information was retained to perform HiLo reconstruction.

### Volumetric imaging using standard LSFM methods with C-DSLM

A 3D scan in standard LSFM mode involved holding the light-sheet and imaging plane constant (zero applied current to ETL-2 and zero applied voltage to the axial galvanometer mirror) while stepping the axial translation piezo stage at pre-calibrated step sizes. It was possible to apply an initial RI matching offset by applying a current to ETL-2 to bring the sample into focus and provide a constant offset throughout the image volume to mimic the commonly found detection objective piezo motor found in the majority of LSFM designs.

A rotating 3D scan, in either standard LSFM mode or C-DSLM mode, involved capturing a volumetric image, rotating the sample a set amount (for example, 90°) and capturing a new scan. In C-DSLM mode, the calibration was re-created for every angle, ensuring the sample remains in focus, while only an initial RI compensation was applied before each scan when running in standard LSFM mode.

### Imaging tiling

For automated tiling, we utilized the OpenStage platform^37^. The user marked the corners of the desired tiling area by moving the stage using the attached controller. The desired overlap (typically 20−30%) was then set. A grid, corresponding to the upper left physical position of each image was then generated. Autofocus imaging was run at each imaging area, ensuring that the initial axial position was the same by returning to the same applied voltage on the slow-axis galvanometer mirror for each new position. Because of local RI variation, it is possible that there are small mismatches in the axial location of adjacent stacks. TeraStitcher was designed to accommodate this mismatch, motivating its application here. By directly writing a compatible file structure to disk during acquisition, we were immediately able to load imaging data into TeraStitcher without additional formatting^39, 40^.

### Image enhancement using modulated excitation beam

In addition to causing first-order defocus, imperfect clearing and RI spatial heterogeneity within a sample also contributed to the broadening of the light-sheet (reducing axial resolution) and increased scattered fluorescence. HiLo reconstruction is a methodology that easily integrates into the C-DSLM design and provided a significant reduction of out-of-focus fluorescence without any additional relay optics or significant post-processing, for example remote focusing or deconvolution. This technique was also more error-resistant than other structured techniques, as inhomogeneous broadening of the excitation pattern led to widespread reconstruction errors in standard structured illumination microscopy algorithms while HiLo was more robust to this effect^41^. We implemented HiLo reconstruction by modulating the laser intensity during the fast-axis sweep that generates the light-sheet. At every axial position, fluorescence from both a uniform light-sheet and a patterned light-sheet were captured and recorded. These images were then computationally combined to extract the in-focus fluorescence. An example HiLo reconstruction is shown in Supplementary Fig. 5.

### Sample mounting

All samples were mounted to a custom pipette tip mount, inspired by previous work from Zeiss Microscopy, OpenSPIM, and OpenSPIN for our specific imaging chamber and RSSM^42, 43^. The end of the pipette tip was plugged with RIMS and 1% agar and a small amount of cyanoacrylate glue holds the tissue specimen in place at the end of the pipette tip. It was critical to minimize the amount of glue utilized to prevent unwanted fluorescence.

### Proteolipid-promoter mice

Transgenic PLP-eGFP mice (6 months, male, *n* = 5) were used to develop the clearing protocol and measurement techniques^21, 22^. PLP-eGFP mice were perfused with 4% paraformaldehyde. For sagittal brain sections, whole brains were incubated in cryoprotectant for 48 h at 4 °C. Free-floating sagittal 30 μm thick sections were taken on a cryostat and placed in PBS. For cleared tissue, PACT was performed according to the protocols outlined by the Gradinaru group^1^.

Brain sections and spinal cords were incubated overnight in 4% acrylamide with 0.25% photo-initiator in 0.1 M PBS in 5 ml conical tubes at 4 °C. The samples were degassed using a homemade chamber to remove nitrogen and rotated at 37 °C for 2 h. We found that complete degassing was a critical step for sample clarity and antibody labeling. After excess hydrogel was removed by washing three times in 0.1 M PBS, tissue sections were placed in 4-8% SDS in 0.1 M PBS in 5 ml conical tubes and rotated at 37 °C for multiple days (timing dependent on size and type of tissue) and directly mounted in index matching solution (RIMS), consisting of forty grams of Histodenz was dissolved in 30 ml of 0.01 M PBS (final concentration of 88% Histodenz w/v). Samples were incubated in RIMS until transparent.

### Rat lung samples

Sprague dawley rats (2 weeks, male *n* = 5, female *n* = 5) were euthanized and the pulmonary vasculature was flushed with 1000 units per ml of heparin and phosphate buffered saline. The trachea was cannulated and the lungs were pressure inflated with 4% paraformaldehyde in PBS at 30 cm H_2_O pressure for 1 h. Lungs were removed and further fixed overnight in 4% paraformaldehyde in PBS. Lung histological sections were generated according to previously published standard protocols^28–30^. For cleared tissue, PACT was performed according to the protocols outlined by the Gradinaru group^1^. Tissue sections were rinsed in 0.1 M PBS for 1 day, followed by overnight incubation in 4% acrylamide with 0.25% photo-initiator in 0.1 M PBS in 50 ml conical tubes at 4 °C. The samples were degassed using a homemade chamber to remove nitrogen and rotated at 37 °C for 5 h. We found that complete degassing was a critical step for samples clarity and antibody labeling. Following removal of excess hydrogel, tissue sections were placed in 8% SDS in 0.1 M PBS in 50 ml conical tubes and rotated at 37 °C for multiple days (timing dependent on size and type of tissue). After multiple rinses in 0.1 M PBS, each tissue section was incubated in primary antibody for 3 days with gentle agitation and daily replacement of the primary antibody buffer. After multiple rinses in 0.1 M PBS, tissue sections were placed in secondary antibody buffer for 3 days with gentle agitation and daily replacement of the second antibody buffer. After thorough rinsing, the tissues were mounted in degassed index matching solution (RIMS) to limit oxygen bleaching of fluorescent antibody labels.

Detailed information on exogenous fluorescent labels used in these studies is provided in Supplementary Note 4.

### HiLo reconstruction

The image processing for HiLo reconstruction follows the general scheme described by Mertz and Kim^18, 41^. In the C-DSLM application of the method, a pair of images was acquired for the reconstruction: a uniform illumination image and a patterned image. The simplest patterned image that can be utilized with HiLo reconstruction was a sinusoidal illumination pattern. The modulation frequency is a parameter required for the reconstruction, which was obtained from an FFT of the structured imaged summed along the non-modulated axis.

A partially demodulated imaged was calculated according to:

1 $$D\left( x \right) = \left| {U\left( x \right) - 2S(x)} \right|$$

where *U* is the uniform image and *S* is the structured image. The reconstructed HiLo image was then obtained from

2 $${I_{{\rm{HiLo}}}}\left( x \right) = \eta \cdot {D_{{\rm{LP}}}}\left( x \right) + {U_{{\rm{HP}}}}\left( x \right)$$

Here, a low-pass filter (LP) was applied to the partially demodulated image and a high-pass filter (HP) was applied to the uniform image. *η* was treated as an adjustable parameter (theoretically equal to *π*/2*M*, where *M* is the modulation amplitude) that was manually adjusted to obtain the clearest image or automatically determined by simultaneously maximizing the SE-DCT and brightness as a function of *η* for a subset of the images within an image stack (Supplementary Fig. 5). In practice, we found using a Gaussian kernel for high-pass filtering to produce the best results.

### Image deconvolution

To investigate the computational efficiency and effectiveness of HiLo reconstruction, we compared the technique to GPU-based deconvolution and CPU-based deconvolution methods. For GPU-based deconvolution, we utilized custom software, based on code provided by Bruce and Butte^44^, to deconvolve images on a custom workstation with two NVidia Titan cards. We found that HiLo reconstruction requires roughly ×2 longer computational time, but GPU deconvolution did not for background from scattering as well as HiLo reconstruction. For CPU-based deconvolution, we evaluated multiple freely available and commercial solutions (SVi, AutoQuant). We found the average computational time for CPU-based deconvolution was at minimum ×20 longer as compared to HiLo reconstruction or GPU-based deconvolution.

### Image registration for tiled volumetric images

We utilized TeraStitcher, a freely available software package, to align and stitch large image datasets together from multiple independent image areas^39, 40^. Overlapping volumes were imaged, stored in a directory structure compatible with TeraStitcher. The images were then aligned using image cross-correlation and tiled. After tiling, a multi-resolution image set, compatible with TeraFly, was stored to disk.

### Image visualization

Vaa3D a freely available software package, was utilized to visualize individual images areas^44^. TeraFly, a freely available plug in within Vaa3D, was used to visualize tiled images produced by TeraStitcher^40^. For 3D image display in this manuscript, the only image filtering applied was a simple threshold to remove low pixel values before rendering.

### Two-dimensional image acquisition and analysis

To visualize the entire sagittal brain section, tiled *z*-stacks were taken on a Leica SP5 confocal microscope using a ×25, NA 0.95 water-immersion objective. PLP-eGFP+ cells were quantified in the entire sagittal brain section using custom MATLAB code. The scale of the images is 0.8 μm per pixel. The acquired *z*-stack was rendered 2D through a maximum intensity projection using the Leica software. Images were analyzed using a custom MATLAB program. First, background subtraction was performed to render the background more homogenous. For PLP-eGFP + cells, the background detection eliminated signal from the PLP-eGFP + oligodendrocyte processes and allowed for a better detection of the signal from the cell bodies. Next, positive pixels were determined using a user-defined threshold value. The threshold value was determined by observing the overlap of the thresholded image and the original image. Connected objects were identified within the positive pixels, correcting for potential multi-cellular objects. Those connected objects that fall within a user determined size for a cell body were counted.

To visualize lung vascular complexity, wide-field bright field images were taken on a Nikon dissecting scope using a ×20 air immersion objective. Vascular complexity was quantified using custom MLI code. Evenly spaced horizontal and vertical grids were independently placed over the acquired images. The chord length was calculated for each image using established MLI techniques^28–30^.

### Three-dimensional image acquisition and analysis

To visualize large physical volumes of PLP-eGFP tissue, axial stacks were generated using the multiple objectives on the C-DSLM. PLP-eGFP+ cells were quantified using the Vaa3D cell counting plugin^45^. We additionally applied this plugin to the 2D confocal images to verify both methodologies produced accurate cell counts. The Vaa3D plugin was solely utilized for light-sheet imaging because it was more memory efficient with large image stacks than MATLAB.

To visualize large physical volumes of distal rat lung, axial stacks were generated using multiple objectives. MLI was performed in three-dimensions using a modified MLI MATLAB code that utilized a three-dimensional grid. Areas outside of the lung were excluded per the MLI protocol. The values reported here were all MLIs found throughout the entire lung, leading to a larger heterogeneity due to the inclusion of distal and proximal lung structures.

For more in-depth analyses such as myelin tracing, we utilized a variety of image analysis techniques. For example, to quantify airway and vascular systems within distal lung imaging and neuron networks within brain imaging, we applied a multi-scale enhancement filter^46, 47^, utilized the *APP2* algorithm to identify and label the network within the image area^48^, manually corrected APP2 network tracing, and then used Vaa3D to annotate features such as individual oligodendrocytes versus myelin tracks in PLP-eGFP brain tissue (Supplementary Fig. 2).

To verify the above method, we manually traced the vascular and airway networks within the distal lung as well as the myelin pencil fibers in the PLP-eGFP cortex^45, 49^. We compared the results to the above semi-automated network tracing. Using the neuron-distance function in Vaa3D, we found that the networks overlap by > 90%. The main source of tracing errors were: (1) connections made between individual network features and cell bodies and (2) multiple endpoints at the edge of network features both of which must be manually curated (Supplementary Fig. 2C).

### Code availability

The microscope is controlled using custom software that integrates Arduino and Python code for all acquisition modes. This code is available upon request.

### Data availability

All imaging data are available through the University of Colorado library system upon request.

## Electronic supplementary material

Supplementary Information

Supplementary Movie 1

Supplementary Movie 2

Supplementary Movie 3

Supplementary Movie 4

Supplementary Movie 5
