# Supplementary material for: Automatic and adaptive heterogeneous refractive index compensation for light-sheet microscopy
Source: Nat Commun. 2017 Sep 20;8:612. doi: 10.1038/s41467-017-00514-7 (PMC5606987; doi:10.1038/s41467-017-00514-7)
Supplement: Supplementary file 1 — Supplementary Information [file 41467_2017_514_MOESM1_ESM.pdf]

File name: Supplementary Information
Description: Supplementary Figures, Supplementary Notes and Supplementary References

File name: Supplementary Movie 1
Description: Three-dimensional rendering of mouse PLPeGFP cortex. Volumetric rendering of a 1.3 mm x 1.3 mm x 5 mm image (scale bar – 500 μm) acquired using a 10x/NA 0.28 aim immersion detection objective. Each axial step is approximately 1 μm. Myelin tracks and individual oligodendrocyte cells are well visualized. Dendrites are visible upon zooming in.

File name: Supplementary Movie 2
Description: Three-dimensional rendering of individual mouse PLPeGFP cells within cortex. Volumetric rendering of a 0.5 mm x 0.5 mm x 2 m image (scale bar – 50 μm) acquired using a 20x/NA 1.0 CLARITY immersion detection objective. Each axial step is approximately 0.4 μm. Individual oligodendrocyte cells and dendrites are well visualized.

File name: Supplementary Movie 3
Description: Three-dimensional rendering of mouse PLP-eGFP spinal cord. Volumetric rendering of a 6 mm x 4 mm x 4 mm tiled image (scale bar – 500 mm) acquired using the 10x/NA 0.28 air immersion detection objective. Long scale myelin tracks, in addition to individual oligodendrocyte cells are well visualized.

File name: Supplementary Movie 4
Description: Three-dimensional rendering of individual mouse PLP-eGFP cells within spinal cord. Volumetric rendering of a 0.5 mm x 0.5 mm x 4 mm image (scale bar – 500 μm) acquired using a 10x/NA 0.28 air immersion objective. Individual oligodendrocyte cells, myelin tracks, and dead space are well visualized.

File name: Supplementary Movie 5
Description: Three-dimensional rendering of distal airspace. Volumetric rendering of a 0.75 mm x 0.75 mm x 0.25 mm image (scale bar – 500 μm) acquired using a 10x/NA 0.28 air immersion objective. Blood vessels (red) and airways (blue) are labeled using immunofluorescence. Alveolar air space is well visualized.

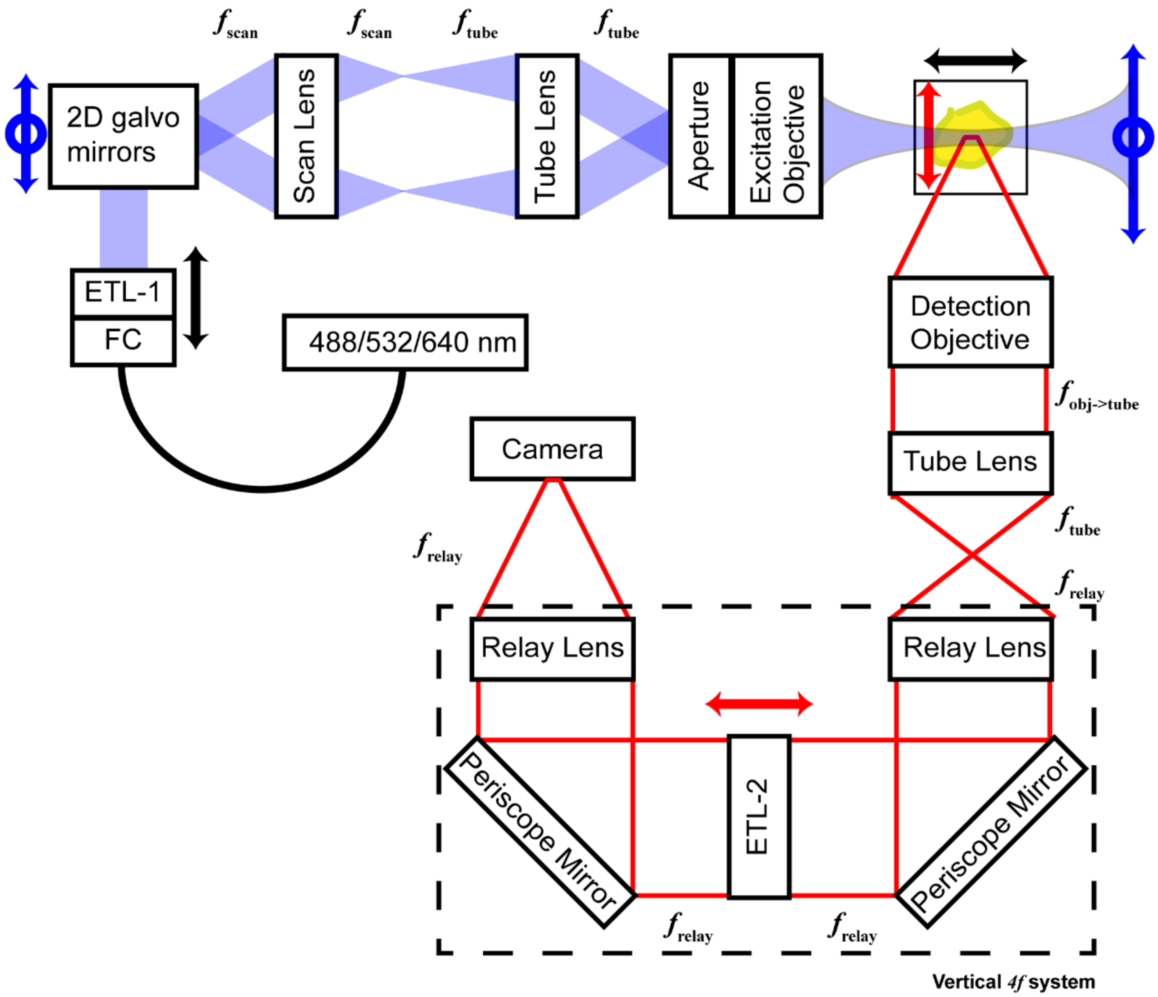

Supplementary Figure 1 – Cleared tissue digital scanned light-sheet microscopy (C-DSLM) optical layout. Excluding sample motion and rotation (*xyzθ*), there are four degrees of freedom within the system. The thinnest area of the exciting Gaussian light-sheet was laterally translated using excitation electro-tunable lens (ETL-1) (black lines with arrows). ETL-1 was placed at a telecentric position to the back aperture of the excitation objective by physically translating the optic along the optical axis between the fiber coupler and the galvanometer mirrors until this position was determined. The physical location of the light-sheet was altered in-plane or axially (blue line with arrows) using the two-dimensional scanning unit. The unit consisted of the scanning mirrors, scan lens (f_{scan} = 70 mm), and tube lens (f_{tube} = 200 mm). The position of the detection plane was altered using a *4f* relay system containing the detection electro-tunable lens (ETL-2). The optimal position of the objective and tube lens were determined for each objective by calculating L:

(1)

Where $\emptyset_{\mathrm{tube}}$ is the entrace pupil of the tube lens, is the exit pupil of the detection objective (DO), F_2 is the focal length of the tube lens, and Ø is the image field diameter of the detector.

The maximum relay lens focal length (f_{relay} = 300 mm) that can be used while maintaining the full NA of the detection objective was determined in a similar manner by calculating $f_{relay,max}$:

(2)

Where $\emptyset_{\mathrm{ETL-2}}$ is the entrance pupil of the ETL placed in the imaging arm and $\emptyset_{\mathrm{DO}}$ is the exit pupil of the detection objective. ETL-2 is placed on an *xyz* translation stage to ensure that the optical axial precisely aligns with the middle of the lens and that the optic is located at the correct plane within the *4f* system.

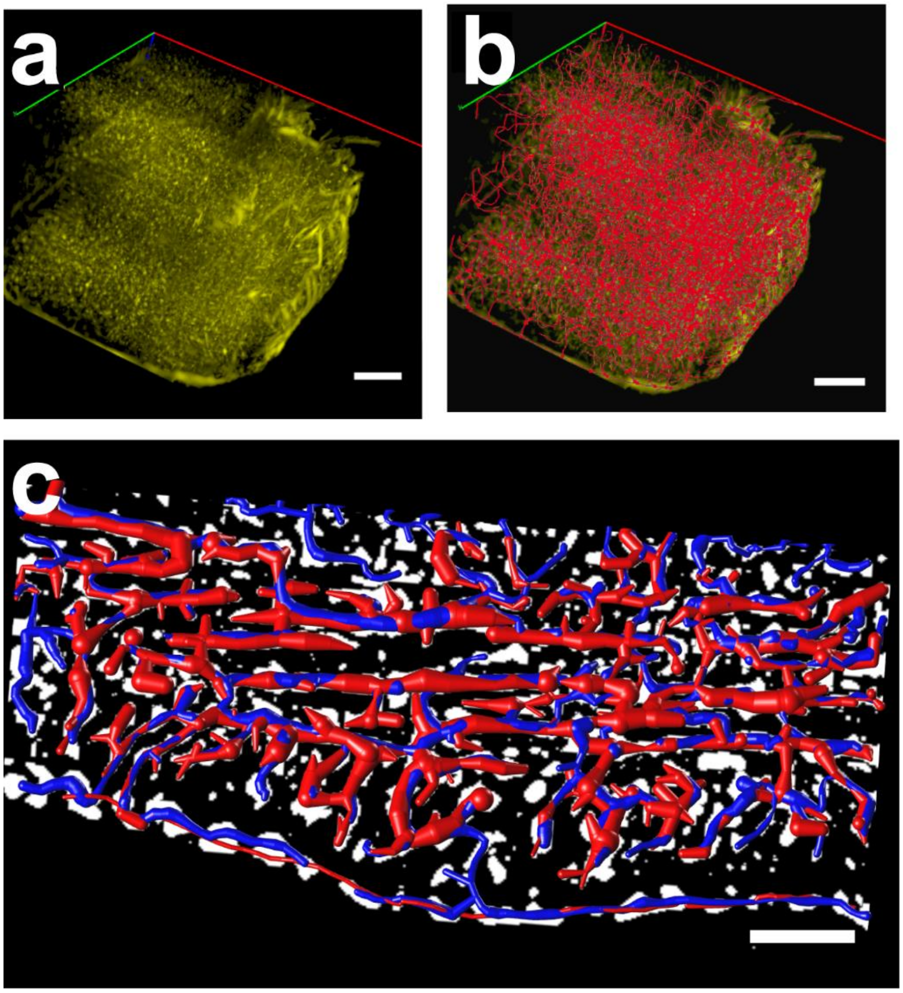

Supplementary Figure 2 –Network quantification in passive CLARITY (PACT)-treated PLP-eGFP mouse spinal cord. a) Maximum intensity projection of tiled multiscale adaptive image filtered cleared tissue digital scanned light-sheet microscopy (C-DSLM) image of PLP-eGFP spinal cord. (scale bars – 250 μm) b) Network structure (red) identified using APP2 and Ultratracer after subtraction of cell bodies and manual curation. (scale bars – 250 μm) c) Manual (blue) and semi-automatic (red) tracing of individual image plane (maximum projection of 5 individual slices, totaling 5 μm). The semi-automatic tracing parameters were optimized based on the distance, calculated using Vaa3D, between the resulting SWC network structures. This procedure was iterated over approximately 25% of total image volume using random sampling (scale bar – 500 μm). These images and analyses are representative of five experiments for spinal cords isolated from different animals.

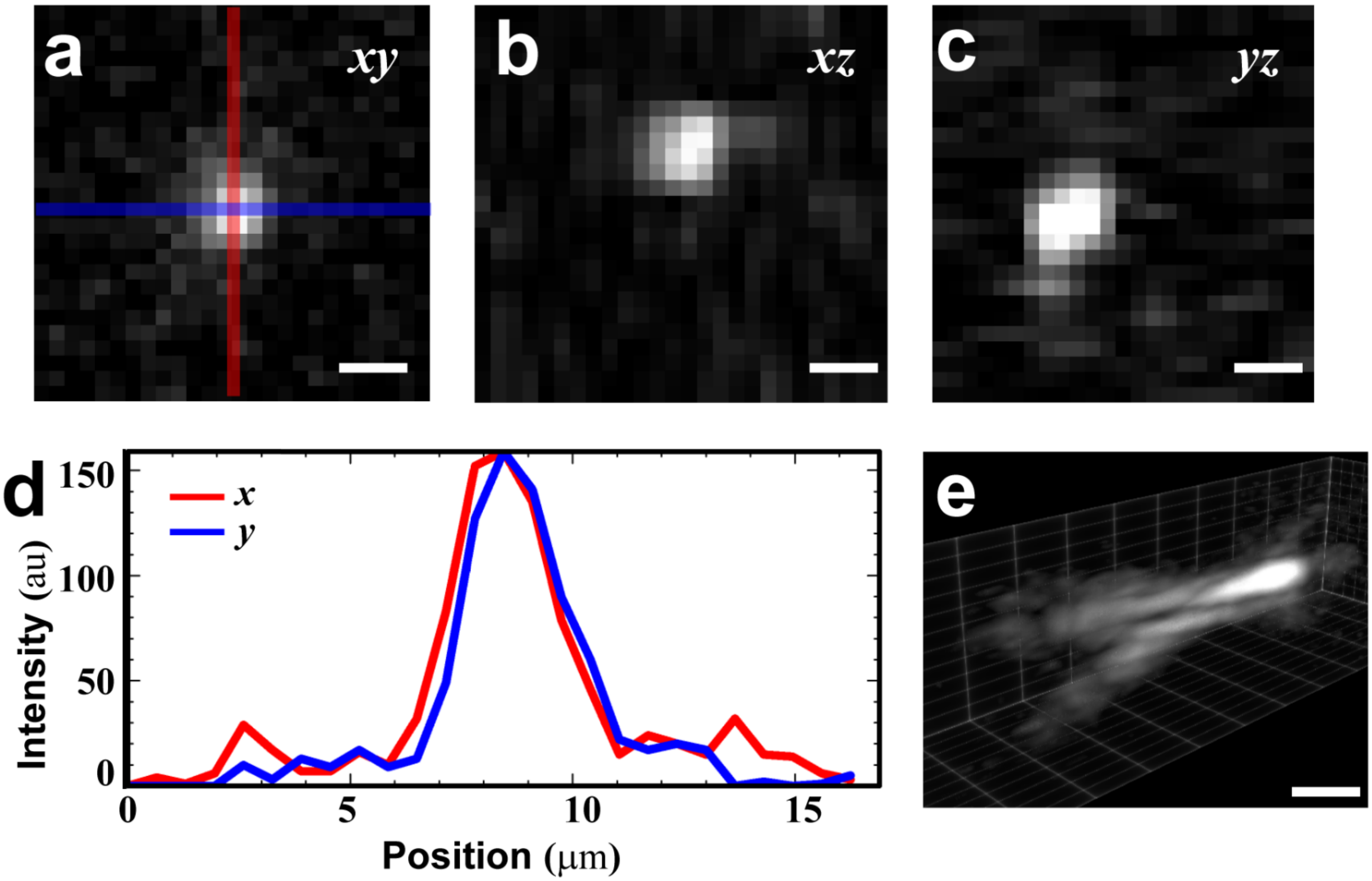

Supplementary Figure 3 – Point spread function (PSF) of cleared tissue digital scanned light-sheet microscopy (C-DSLM) using the 10× detection objective (DO). A) *xy* projection of an individual TetraSpeck bead excited using the 488-nm laser. The intensity profiles for the red and blue lines are given below in (e) (scale bar - 1200 nm). b) *xz* and c) *yz* projections of the same spot (scale bar - 1200 nm). Spherical aberration is clearly present, as there are strong side-lobes. This is visible in the 3D rendering of the PSF given in (f). d) Intensity profiles across the bead shown in (a). Fitting a PSF constructed from averaging thousands of beads in each channel yields a minimum PSF width of 1235 nm for the 488 nm laser. The theoretical minimum spot size, calculated using the Gibson-Lanni model for 10×/NA 0.28 objective is 871 nm. e) We observe classic spherical aberration PSF pattern when many beads are averaged and rendered in 3D (scale bar - 1200 nm).

a b

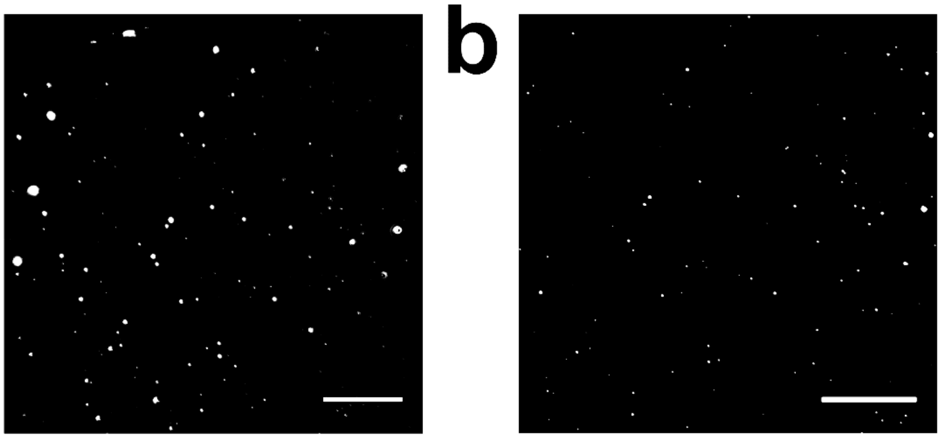

Supplementary Figure 4 – Uneven focus across field-of-view due to small misalignment in the detection electro-tunable lens (ETL-2). Even when the initial alignment of the grid target is near perfect for each objective, it is necessary to perform fine alignment of both objective position and the *4f* system containing ETL-2. a) For this image, many individual TetraSpeck beads are in focus, but there is clear spatial inhomogeneity in the focus. This is only detectable and correctable by fine-tuning using a bead calibration sample. b) After alignment of the periscope mirrors and ETL-2 *xyz* position, we obtain a flat field of view. The PSF of individual beads can be checked at each corner to ensure similar results are obtained as in Supplementary Figure 3 (scale bar – 250 μm).

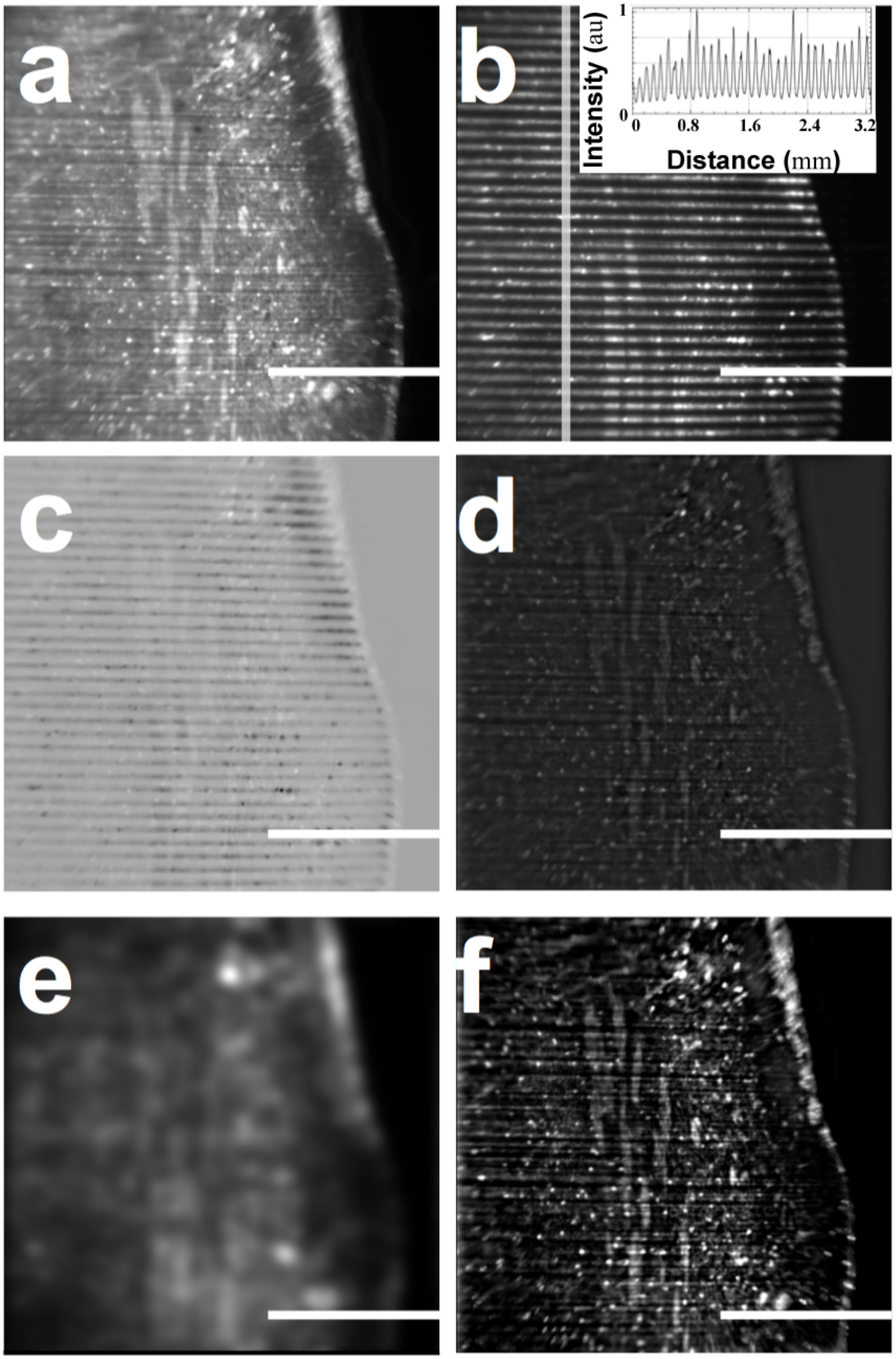

Supplementary Figure 5 – HiLo background subtraction procedure for one slice from PLP-eGFP spinal cord C-DSLM image set. The formulas and calculation steps are given in the methods section. a) Uniform illumination image, $U(x,y)$, generated by rapid oscillation of the in-plane scanning mirror while the frame is exposing, taking into account the rolling shutter of the sCMOS detector. b) Structured image, $S(x,y)$, generated by modulating the laser beam and digital patterning of the in-plane scanning mirror while the frame is exposing. Inset: plot profile of vertical line cut, showing the sinusoidal pattern. c) Partially demodulated image, $D(x,y)$. d) Hi-pass filtered uniform illumination image, $U_{HP}(x,y)$. e) Lo-pass filtered demodulated image, $D_{LP}(x,y)$. f) Final HiLo image, $I_{HiLo}(x,y)$, using an η parameter of 0.3 for this image. (all scale bars – 500 μm)

Supplementary Note 1

Imaging depth of field dependence on magnification and numerical aperture. In the classic light-sheet fluorescence microscope (LSFM) design, the location of the light-sheet is fixed along the optical axis of the detection arm such that the imaging plane and light-sheet are coplanar. The sample position is mechanically manipulated within the fixed focus region to obtain images at various depths. Translating the sample results in imaging through a variable combination of refractive index (RI). As a consequence, the imaging focal plane shifts by an approximately amount $\mathrm{RI}_{\mathrm{total}} \times \Delta z_{\mathrm{sample}}$, which introduces an offset between the fixed light-sheet and the imaging plane. This mismatch between the excitation and detection plane is present in all LSFM designs. However, many LSFMs are designed to trade-off magnification (M) and numerical aperture (NA) to *maintain* a large depth-of-field (d_{tot}) such that the light-sheet is always located in the detection volume,[1] utilize one of an entirely new class of custom microscope objectives that either matches the buffer RI or accommodates a variable RI, or optically translate the light-sheet and physically translate the detection objective (remote focusing).[2–10] Many commercial LSFM designs that currently advertise compensation for specific optical clearing techniques through RI compensation typically do so with a constant offset of approximately $(1-\mathrm{RI}_{\mathrm{sample}})\times\Delta z_{\mathrm{sample}}$, assuming that the RI remains constant throughout the sample and imaging media.

The selection of M and the detection objective NA determine d_{tot} and to achieve higher in-plane resolutions in LSFM measurements, the M and NA of the detection arm must be increased. For moderate NA (0.1-0.5) detection objectives, d_{tot} consists of a trade-off between the diffraction limited (approximately NA^{-2}) and lateral resolution (approximately NA^{-1}) terms at a given wavelength (λ), refractive index (n), and the smallest distance that can be resolved by the detector (e).

$$d_{\mathrm{tot}} = \frac{\lambda \cdot n}{\mathrm{NA}^2} + \frac{n}{M \cdot \mathrm{NA}} e \quad \textbf{(3)}$$

Supplementary Note 2

Effective displacement of the detection electro-tunable lens (ETL-2). Because the excitation and detection arm of the C-DSLM are independent, the instrument can compensate for focal shifts by adjusting the focal length of the detection arm with ETL-2. The effective displacement ($\Delta z_{\mathrm{ETL-2}}$) is dependent on the magnification of the detection objective (M_{DO}), the RI of the imaging medium (n), the focal length of relay lenses (f_{L1}), and the focal length of the ETL-2 ($f_{\mathrm{ETL-2}}$)

$$Dz_{ETL-2} = -\frac{1}{M_{DO}^2}\left[\frac{n \cdot f_{L1}^2}{f_{ETL-2}}\right]$$

$$\Delta z_{\mathrm{ETL-2}} = -\frac{1}{M_{\mathrm{DO}}^2}\left[\frac{n \cdot f_{\mathrm{L1}}^2}{f_{\mathrm{ETL-2}}}\right] \quad \textbf{(4)}$$

In theory, these equations and the response curve of the ETL-2 can be used to encode the required hardware settings for any given sample RI. However, we find imaging specific calibration planes and fine-tuning the hardware is the best approach to ensure the focal plane of the detection arm coincides with the axial location of the light-sheet because the index of refraction, *n*, in this equation is not constant.

Supplementary Note 3

Detailed initial cleared tissue digital scanned light-sheet microscopy (C-DSLM) alignment. After C-DSLM construction and alignment of the scanning mirrors and scan lens, it is necessary to align the *4f* configuration to ensure that the detection arm is truly telecentric and directed through the center of the detection electro-tunable lens (ETL-2). We perform alignment using two calibration samples, a grid target for coarse alignment and a microsphere bead sample suspended in agar for fine alignment.

Grid array (rough alignment)

We place a grid target (Edmund Optics) with spacing of 1 mm between lines at the working distance of the detection objective and back illuminate it with a light emitting diode (LED) white-light source. We then manually displace the grid target using the automated stage, refocusing using ETL-2, and compare the magnification grid using the correct pixel size for a given detection objective magnification (e.g. 6.5 μm/10 = 0.65 μm for 10x). To correct for errors, we independently adjust the spacing between the tube lens (TL), first relay lens (L1), ETL-2, and second relay lens (L2) to achieve telecentric alignment of the 4*f* system. Once this has been achieved, we apply a slow sinusoidal signal (0.25 Hz) to ETL-2 across the entire focal range of the lens and adjust the two alignment mirrors to remove tilt and distortion at the camera detection plane due to misalignment of the mirrors and ETL-2. Finally, we measure the distortion of the grid by displacing the grid, re-focusing using ETL-2, and then fine adjusting the alignment mirrors to minimize distortion. This distortion is due to the light not passing through the middle of ETL-2 and any tilt in the grid target. While one can achieve near perfect alignment during this procedure, it is difficult to perfectly align the exciting light-sheet and focal plane of the detector

during an actual experiment due to slight sample chamber tilt and RI mismatch as seen in the next alignment step. This alignment must be repeated for each objective to determine to the best position of the objective between the sample chamber and tube lens. Once determined, these positions are fixed and we find are highly repeatable when changing detection objectives.

Fluorescent TetraSpeck microspheres suspended in RIMS and 1% agar (fine alignment of the optical transfer function)
100 nM of fluorescent TetraSpeck microspheres (Life Technologies) are suspended in a mix of refractive index matching solution (RIMS) and 1% agar that is vortexed, let cool into a column within a glass tube, and finally glued to the end of the 200 μL pipette tip. Once this mixture has cooled, the sample is mounted onto the rotating sample stage mount (RSSM), and the focal position of the light-sheet is set identically for both the 488, 532, and 640 nm lasers using the excitation electro-tunable lens (ETL-1). Ensuring that the cuvette surfaces are perpendicular to both the excitation and detection objective is critical for this step and we utilize the fine manual adjustment of the rotation stage to minimize the distortion in the light-sheet before proceeding to imaging.

Using a multi-band emission filter (Semrock), we locate an area of isolated 0.2 μm TetraSpeck microspheres and apply a slow sinusoidal signal (0.25 Hz) to ETL-2 over a range large enough to axially traverse the axial extent of the microspheres. We utilize the two steering mirrors to obtain nearly symmetric PSFs in both colors given the spherical aberration of system.

Finally, we manually calibrate an image stack and scan through the entire focal range of ETL-2 for each color to check the calibration over the entire range. This procedure is repeated until the field of view is nearly uniform for all visible beads in each focal stack.

Fluorescent FocalCheck microspheres suspended in RIMS and 1% agar (fine alignment of chromatic aberration)
100 nM of fluorescent FocalCheck microspheres (Life Technologies) are suspended in a mix of RIMS and 1% agar that is vortexed, let cool into a column within a glass tube, and finally glued to the end of the 200 μL pipette tip. Once this mixture has cooled, the sample is mounted onto the RSSM, and the focal position of the light-sheet is set identically for both the 488, 532, and 640 nm lasers using ETL-1. Ensuring that the cuvette surfaces are perpendicular to both the excitation and detection objective is critical for this step and we utilize the fine manual adjustment of the rotation stage to minimize the distortion in the light-sheet before proceeding to imaging.

Using a multi-band emission filter (Semrock), we locate an area of isolated microspheres and apply a slow sinusoidal signal (0.25 Hz) to ETL-2 over a range large enough to axially traverse the size of the 15 μm FocalCheck microspheres. We utilize the two steering mirrors to ensure the fluorescence in the 488 nm channel forms a perfect ring around the fluorescence in the 640 nm channel. We similarly iterate for each pair of 488, 532, and 640 nm lasers.

Finally, we manually calibrate an image stack and scan through the entire focal range of ETL-2 for each color to check the calibration over the entire range. This procedure is repeated until chromatic aberrations are minimized across the full range of the focal stack.

Supplementary Note 4

Endothelial cell labeling:
Primary label: biotinylated griffonia Lectin (IB4) Vector #B-1205 lot ZB1017 at 1:100 concentrations.
Secondary label: Alexa488 conjugated streptavidin. Jackson Immunoresearch #016-540-084 lot 123867 at 1:200 concentrations.
Verification for primary label in reference [11].

Cytokertain-18 labeling:
Primary antibody: cytokeratin-18, Thermo Fisher/Invitrogen #PA5-28279 lot RB2149421F at 1:100 concentrations.
Secondary antibody: Alexa647, Invitrogen/Molecular Probes #A31573 lot 439377 at 1:200 concentrations.
Verification for primary antibody in reference [12].

Supplementary References

1. Becker, K., Jährling, N., Saghafi, S. & Dodt, H.-U. Ultramicroscopy: light-sheet-based microscopy for imaging centimeter-sized objects with micrometer resolution. *Cold SpRIng Harb. Protoc.* **2013,** 704–713 (2013).
2. Tomer, R. *et al.* SPED Light Sheet Microscopy: Fast Mapping of Biological System Structure and Function. *Cell* **163,** 1796–1806 (2015).
3. Masson, A. *et al.* High-resolution in-depth imaging of optically cleared thick samples using an adaptive SPIM. *Sci. Rep.* **5,** 16898 (2015).
4. Chmielewski, A. K. *et al.* Fast imaging of live organisms with sculpted light sheets. *Sci. Rep.* **5,** 9385 (2015).

5. Hedde, P. N. & Gratton, E. Selective plane illumination microscopy with a light sheet of uniform thickness formed by an electrically tunable lens. *Microsc. Res. Tech.* n/a-n/a (2016). doi:10.1002/jemt.22707
6. Hedde, P. N. & Gratton, E. Active focus stabilization for upright selective plane illumination microscopy. *Opt. Express* **23,** 14707–14714 (2015).
7. Keller, P. J. & Ahrens, M. B. Visualizing Whole-Brain Activity and Development at the Single-Cell Level Using Light-Sheet Microscopy. *Neuron* **85,** 462–483 (2015).
8. Chen, B.-C. *et al.* Lattice light-sheet microscopy: Imaging molecules to embryos at high spatiotemporal resolution. *Science* **346,** 1257998 (2014).
9. Silvestri, L., Sacconi, L. & Pavone, F. s. Correcting spherical aberrations in confocal light sheet microscopy: A theoretical study. *Microsc. Res. Tech.* **77,** 483–491 (2014).
10. Dean, K. M. *et al.* Diagonally Scanned Light-Sheet Microscopy for Fast Volumetric Imaging of Adherent Cells. *Biophys. J.* **110,** 1456–1465 (2016).
11. Factor for Retinal Neurons and a Critical Neuroprotectant during the Adaptive Response to Ischemic Injury, Am J Path **171**, 53-67 (2007).
12. Antibodypedia verification found at: http://www.antibodypedia.com/gene/271/KRT18/antibody/624264/PA5-28279
